# Wilson disease (novel *ATP7B* variants) with concomitant *FLNC*-related cardiomyopathy

**DOI:** 10.1038/s41439-024-00283-y

**Published:** 2024-08-29

**Authors:** Takeshi Imai, Satomi Mitsuhashi, Kenji Isahaya, Soichiro Shibata, Yosuke Kawai, Yosuke Omae, Katsushi Tokunaga, NCBN Controls WGS Consortium, Hatsue Ishibashi-Ueda, Tsutomu Tomita, Michio Noguchi, Ayako Takahashi, Yu-ichi Goto, Sumiko Yoshida, Kotaro Hattori, Ryo Matsumura, Aritoshi Iida, Yutaka Maruoka, Hiroyuki Gatanaga, Akihiko Shimomura, Masaya Sugiyama, Satoshi Suzuki, Kengo Miyo, Yoichi Matsubara, Akihiro Umezawa, Kenichiro Hata, Tadashi Kaname, Kouichi Ozaki, Haruhiko Tokuda, Hiroshi Watanabe, Shumpei Niida, Eisei Noiri, Koji Kitajima, Yosuke Omae, Reiko Miyahara, Hideyuki Shimanuki, Yosuke Kawai, Katsushi Tokunaga, Yoshihisa Yamano

**Affiliations:** 1 Department of Neurology, St Marianna University School of Medicine, Kawasaki, Kanagawa Japan; 2 Genome Medical Science Project, National Center for Global Health and Medicine, Tokyo, Japan; 3 NCVC Biobank, National Cerebral and Cardiovascular Center, Suita, Osaka Japan; 4 Medical Genome Center, National Center of Neurology and Psychiatry, Kodaira, Tokyo Japan; 5 Department of Bioresources, Medical Genome Center, National Center of Neurology and Psychiatry, Kodaira, Tokyo Japan; 6 Department of Clinical Genome Analysis, Medical Genome Center, National Center of Neurology and Psychiatry, Kodaira, Tokyo Japan; 7 NCGM Biobank, National Center for Global Health and Medicine, Shinjuku-ku, Tokyo Japan; 8 AIDS Clinical Center, National Center for Global Health and Medicine, Shinjuku-ku, Tokyo Japan; 9 Department of Viral Pathogenesis and Controls, Research Institute, National Center for Global Health and Medicine, Ichikawa, Chiba Japan; 10 Center for Medical Informatics and Intelligence, National Center for Global Health and Medicine, Shinjuku-ku, Tokyo Japan; 11 National Center for Child Health and Development, Setagaya-ku, Tokyo Japan; 12 Center for Regenerative Medicine, National Center for Child Health and Development, Setagaya-ku, Tokyo Japan; 13 Department of Maternal-Fetal Biology, National Center for Child Health and Development, Setagaya-ku, Tokyo Japan; 14 Department of Genome Medicine, National Center for Child Health and Development, Setagaya-ku, Tokyo Japan; 15 Research Institute, National Center for Geriatrics and Gerontology, Obu, Aichi Japan; 16 Central Biobank, National Center Biobank Network, Shinjuku-ku, Tokyo Japan; 17 Genome Medical Science Project (Toyama), Research Institute, National Center for Global Health and Medicine, Shinjuku-ku, Tokyo Japan

**Keywords:** Genetic testing, Genetics of the nervous system

## Abstract

We report a case of Wilson disease (WD) with dilated cardiomyopathy in which whole-genome sequencing (WGS) revealed the rare co-occurrence of two novel compound heterozygous *ATP7B* pathogenic variants (NM_001005918.3:c.2250del/p.N751Tfs*9 and c.3496C>T/p.L1166F) and a known FLNC pathogenic variant. Our results highlight the usefulness of WGS, even in the diagnosis of well-characterized genetic diseases such as WD.

Wilson disease (WD) is an autosomal recessive neurodegenerative disorder affecting copper metabolism (MIM #277900) via pathogenic variants of the *ATP7B* gene. The mutated gene prevents proper function of the copper transport protein, leading to copper accumulation in the liver, brain, kidneys, and skeletal system^1^. Notably, pathogenic variants that eliminate *ATP7B* tend to result in more severe and specific phenotypes than missense variants^2,3^.

The clinical features of WD are highly variable. Although most symptomatic patients present with hepatic and/or neuropsychiatric dysfunction, WD can affect almost any organ in the body, including the heart, resulting in a very complex clinical profile in individuals^4^. The variety of cardiac symptoms observed in some WD patients include arrhythmia, cardiomyopathy, and autonomic dysfunction. Structural and functional changes in the heart in WD patients have been reported to manifest primarily as mild left ventricular hypertrophy and diastolic functional changes, which are more apparent in patients with neurological WD^5^. WD patients with severe clinical manifestations may present with cardiac symptoms and are at greater risk for developing structural heart disease^6^. However, the possibility of complications of other genetic diseases that may be masked by the diverse clinical manifestations of WD patients should receive more attention. Here, we report a rare case of WD with *FLNC*-related cardiomyopathy.

A 50-year-old woman visited another clinic due to a 20-year history of exertional shortness of breath. Echocardiography revealed a dilated left ventricular (LV) end-diastolic diameter (65 mm), a dilated LV end-systolic diameter (55 mm), a diffusely hypokinetic left ventricle with a depressed ejection fraction (45–50%), and severe mitral valve regurgitation. Coronary angiography revealed no coronary artery stenosis. Although cardiovascular MRI and myocardial scintigraphy were not performed to assess the cardiac lesions, the patient was clinically diagnosed with dilated cardiomyopathy (DCM). Despite optimal medical therapies, the functional status of her DCM deteriorated (ejection fraction, 30–35%), and she underwent mitral valve repair at our hospital.

At 58 years of age, the patient noticed shaking in her hands and consulted a neurologist. During the neurological examination, she exhibited postural and kinetic tremors in her upper limbs. By age 68, she struggled with tasks such as opening or closing a water tap, operating a TV remote control/smartphone, and brushing her teeth. Additionally, she exhibited rigidity in all her limbs, leading to her admission to the neurological department. The patient’s tremors and rigidity were mild and did not significantly impair movement. However, she was unable to explain how to use necessities of daily living and could not use them correctly. Tests revealed low ceruloplasmin (8.8 mg/dL; reference range, 30–58 mg/dL) and copper (21 µg/dL; reference range, 70–140 µg/dL) blood concentrations and high urine copper excretion (2985 µg/day; reference range, 15–60 µg/day; 2000 mL urine). An ophthalmologist observed bilateral Kayser-Fleischer rings, and an MRI of the brain showed hyperintensity in the white matter, which is atypical in WD, and in the basal ganglia (Fig. 1A–C). Based on these findings, the patient was diagnosed with WD. While no family history of WD or other neurological or psychiatric disorders was reported, the patient’s son died at age 30 due to DCM, although medical details are unknown. Symptoms of WD and DCM developed earlier than the relatively late onset of neuropsychiatric symptoms, and the family history of DCM in the patient’s son prompted us to perform whole-genome sequencing (WGS) to diagnose the patient (Fig. 2A).

**Fig. 1:**
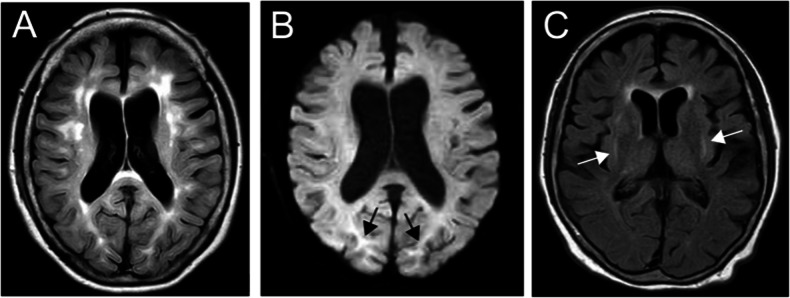
Clinical and radiological features of the patient. **A** FLAIR image showing periventricular hyperintensity and deep and subcortical white matter hyperintensity (axial; 1.5 T; TR, 8000 ms; TE, 100 ms). **B** DWI showing high-intensity signals (arrows) in the region of the corticomedullary junction in the bilateral occipital lobes (axial; 1.5 T; TR, 3732 ms; TE, 89 ms). **C** FLAIR image showing basal ganglia hyperintensity (axial; 1.5 T; TR, 8000 ms; TE, 100 ms).

**Fig. 2:**
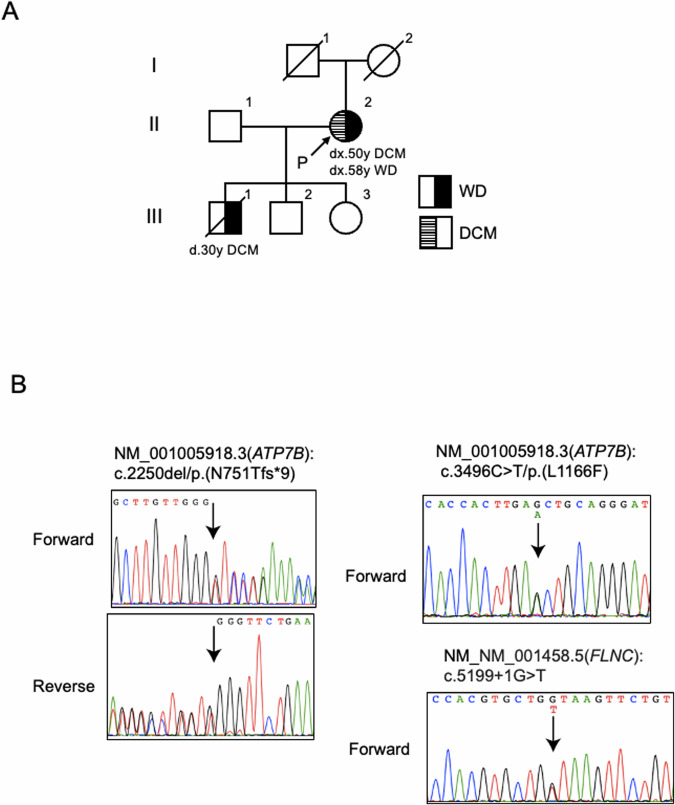
Family pedigree of the patient and Sanger sequencing electrophoretograms. **A** Family pedigree of the patient. The proband’s mother died at the age of 74 due to pancreatic cancer. The father died at the age of 54 due to unknown causes. The patient’s son died at the age of 30 due to DCM. **B** Sanger sequencing confirmed these variants.

WGS and variant filtering were performed as described previously^7^. DNA samples were obtained from the patient’s peripheral blood using a QIAamp® DNA Blood Midi/Maxi kit (Qiagen), and DNA libraries were prepared with the TruSeq DNA PCR-Free HT Library Prep Kit with a 550-bp insertion size. WGS was conducted using an Illumina NovaSeq 6000 instrument at Takara Bio Laboratory. Data processing was carried out using Parabricks^®^ Pipeline version 3.5.0 (NVIDIA Clara™) at Takara Bio Inc., as previously described^7^. Variant annotation was conducted using ANNOVAR (https://annovar.openbioinformatics.org/en/latest/). WGS revealed heterozygous variants, NM_001005918.3:c.2250del/p.(N751Tfs*9) and c.3496C>T/p.(L1166F), in *ATP7B* responsible for WD. Sanger sequencing confirmed these variants (Fig. 2B). The variants were confirmed to be in trans by long-read sequencing (Supplementary Methods and Supplementary Fig. 1), suggesting that the WD patient was a compound heterozygote. Neither variant was reported in reference population databases or disease-related databases. NM_001005918:c.3496C>T was predicted to impair protein function according to in silico analyses. According to the guidelines of the American College of Medical Genetics and Genomics and the Association for Molecular Pathology (ACMG/AMP guidelines)^8^, we classified NM_001005918.3:c.2250del as “pathogenic” (PVS1, PM2, and PP4) and NM_001005918.3:c.3496C>T as “likely pathogenic” (PM2, PM3, PP3, and PP4). Therefore, the patient was determined to be a compound heterozygote with two different novel pathogenic variants in *ATP7B*. In addition, we identified a heterozygous canonical splice site variant, NM_001458.5:c.5199+1G>T, potentially resulting in protein truncation, in *FLNC*, which is responsible for cardiomyopathy (MIM #617047) and muscular diseases (MIM #609524 and #614065). DNA from the patient’s deceased son, who succumbed to DCM at the age of 30 years, was unavailable. This variant was rarely observed in population cohorts in the Genome Aggregation Database (gnomAD, 0.0009%) and was not found in the WGS database of 9850 Japanese control individuals^7^. Therefore, this variant can be classified as “likely pathogenic” (PM1, PM2 supportive, PP3, PP4, and PP5) according to the ACMG/AMP guidelines^8^. This variant was also reported as likely pathogenic by two submitters in ClinVar.

In conclusion, we reported a case illustrating the exceptionally rare co-occurrence of WD and *FLNC*-related cardiomyopathy. This report emphasizes the importance of comprehensive gene analysis, even in the diagnosis of well-characterized genetic diseases such as WD.

## HGV database

The relevant data from this Data Report are hosted at the Human Genome Variation Database at 10.6084/m9.figshare.hgv.3403, 10.6084/m9.figshare.hgv.3406, 10.6084/m9.figshare.hgv.3409

## Supplementary information

Supplementary Data

Supplementary Figure 1
