# Supplementary material for: Wilson disease (novel ATP7B variants) with concomitant FLNC-related cardiomyopathy
Source: Hum Genome Var. 2024 Aug 29;11:34. doi: 10.1038/s41439-024-00283-y (PMC11362149; doi:10.1038/s41439-024-00283-y)
Supplement: Supplementary file 1 — Supplementary Data [file 41439_2024_283_MOESM1_ESM.docx]

***Supplementary data***

**Methods**

Cas9 enrichment sequencing of the two variants in *ATP7B* was performed as previously described using CRISPR/Cas9 system and nanopore long read sequencing^1^. We designed two guide RNAs targeting the upstream and downstream regions of the target sequence (chr13:51935462-51946629), which encompass both ATP7B variants. The upstream and downstream guide RNAs are as follows: 5’-ATGCACACCAGGCTCCATGT-3’ and 5’-CAGTAACGTGTTCTCTATGA-3’, respectively.

The custom Alt-R CRISPR-Cas9 guide RNA design tool (IDT) was used to design guide RNAs. Nanopore library preparation was performed using SQK-CS9109 (Oxford Nanopore Technologies) following the manufacturer’s protocol and a method previously described by Tachikawa et al^1^.

CRISPR/Cas9-cleaved DNA fragments were ligated with adaptors for R10.4 nanopores (SQK-LSK114) and subjected to nanopore sequencing using a MinION R10.4 flowcell (FLO-MIN114, Oxford Nanopore Technologies) on the GridION nanopore platform (Oxford Nanopore Technologies). Long reads obtained were aligned to hg38 as described here (https://gitlab.com/mcfrith/last/-/blob/main/doc/last-cookbook.rst) or elsewhere ^1^.

**Figure legend for Supplementary figure 1**

Reads encompassing the two ATP7B variants, c.2250del: p.(N751Tfs*9) (right) and c.3496C>T: p.(L1166F) (left). In total, 65 reads were obtained by Cas9 enrichment sequencing. Reads were segregated based on the c.3496C>T:p.(Leu1166Phe) variant and visualized using IGV software (ver. 2.14.0). Each variant is present on different reads, confirming compound heterozygosity.

**Reference**

1. Tachikawa K, Shimizu T, ImaiT, Ko R, Kawai Y, Omae Y et al. Cost-Effective Cas9-Mediated Targeted Sequencing of Spinocerebellar Ataxia Repeat Expansions. J Mol Diagn 2023 Nov 24:S1525-1578(23)00272-6. doi: 10.1016/j.jmoldx.2023.10.004. Online ahead of print.
