# Supplementary figures and images for: Wilson disease (novel ATP7B variants) with concomitant FLNC-related cardiomyopathy
Source: Hum Genome Var. 2024 Aug 29;11:34. doi: 10.1038/s41439-024-00283-y (PMC11362149; doi:10.1038/s41439-024-00283-y)

# Supplementary Figure 1

chr13:51946473:G:del  
c.2250del: p.(N751Tfs\*9)

Allele 1

Allele 2

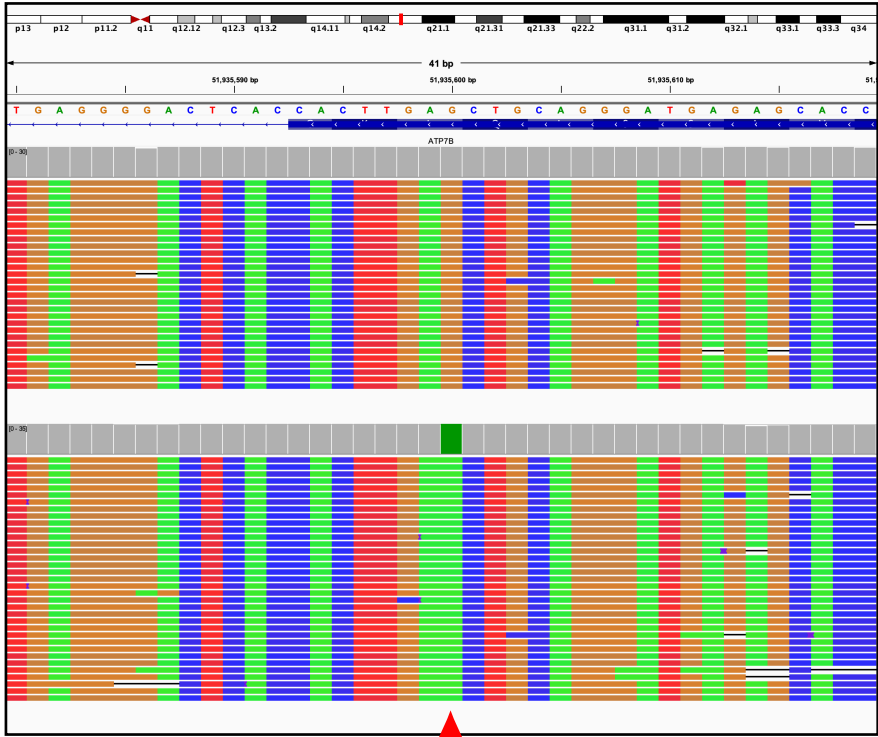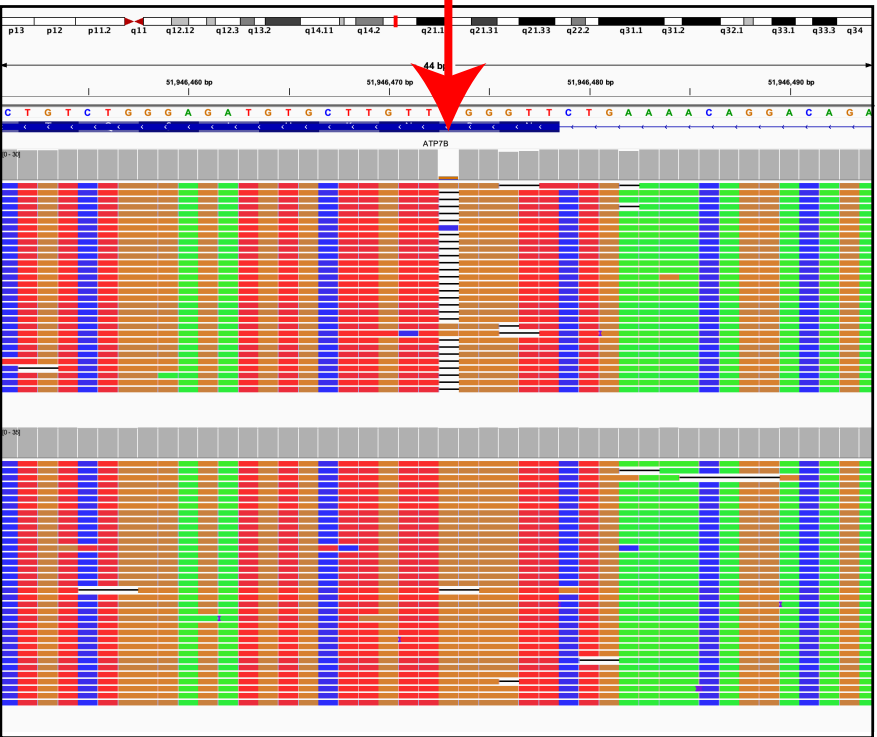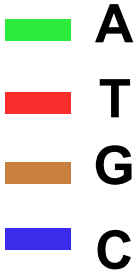

Supplement: Supplementary file 2 — Supplementary Figure 1 [file 41439_2024_283_MOESM2_ESM.pdf]
